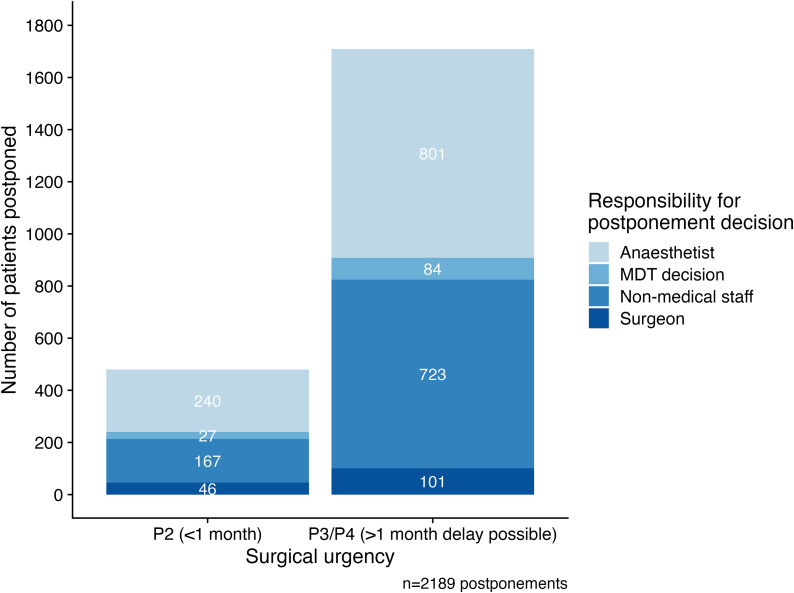# The Postponement and Cancellations in Elective Care study: a national evaluation of case postponements and cancellations in elective surgical pathways

**DOI:** 10.1016/j.bja.2026.01.046

**Published:** 2026-04-24

**Authors:** James Bedford, Emma McCone, Adam Hunt, Eleanor Warwick, Iain Moppett, Martha Belete, Laura Cortes, Sarah Hayden, Andrew Kane, Justin Kua, Emira Kursumovic, Georgina Singleton, James Young, Adam Foster, Judith Hendley, Laura Saucedo-Cuevas, S. Ramani Moonesinghe, Joanna Simpson

**Affiliations:** 1 National Institute for Health Research–Central London Patient Safety Research Collaboration, London, UK; 2 Department of Anaesthesia & Perioperative Medicine, University College London Hospitals NHS Foundation Trust, London, UK; 3 Centre of Perioperative Medicine, Research Department for Targeted Intervention, Division of Surgery and Interventional Science, University College London, London, UK; 4 Getting It Right First Time Programme, NHS England, London, UK; 5 Directorate of Perioperative and Critical Care Medicine, The Newcastle upon Tyne Hospitals NHS Foundation Trust, Newcastle-upon-Tyne, UK; 6 Department of Anaesthesia, Lewisham & Greenwich NHS Trust, London, UK; 7 Centre for Research & Improvement, Royal College of Anaesthetists, London, UK; 8 Faculty of Medicine & Health Sciences, University of Nottingham, Nottingham, UK; 9 Department of Anaesthesia, South Tees Hospital NHS Foundation Trust, Middlesbrough, UK; 10 Department of Anaesthesiology, St Paul’s Hospital, Vancouver, BC, Canada; 11 Department of Anaesthesia, Cambridge University Hospital NHS Foundation Trust, Cambridge, UK; 12 Elective Recovery Programme, NHS England, London, UK; 13 Department of Anaesthesia, East Suffolk and North Essex NHS Foundation Trust, Colchester, UK

**Keywords:** elective surgery, NHS, preoperative assessment, surgery cancellation, surgery postponement

## Abstract

**Background:**

Elective surgical postponements and cancellations adversely affect patient experience, clinical outcomes, and theatre efficiency within the UK NHS. The Postponement and Cancellations in Elective Care study aimed to establish the national incidence and underlying causes of elective surgical postponements at preoperative assessment and cancellations occurring within 24 h of planned surgery.

**Methods:**

This 7-day prospective service evaluation (November 11–18, 2024) collected data from participating NHS England trusts *via* an online platform. Preoperative assessment postponements were defined as any factor preventing a patient with a ‘to come in’ date from undergoing surgery as planned, or preventing preoperative assessment completion as ‘good to go’ within 2 weeks for those without a ‘to come in’ date. Cancellations were defined as a decision not to proceed within 24 h of planned surgery.

**Results:**

Seventy-eight trusts provided complete incidence data covering 22 573 preoperative assessment appointments and 19 905 planned procedures. The national preoperative assessment postponement incidence was 8.7% (95% confidence interval, 6.9–10.8%), most commonly attributable to further investigation or tests (*n*=595, 27.2%). The national cancellation incidence was 9.9% (95% confidence interval, 8.7–11.3%), most frequently associated with acute medical conditions (*n*=515, 23.8%) and list overruns (*n*=315, 14.5%).

**Conclusions:**

The Postponement and Cancellations in Elective Care study provides the first national overview of elective surgical pathway disruption in the UK. Nearly half of postponements were attributable to requirements for additional investigation or specialty review, indicating the importance of earlier optimisation and robust preoperative assessment fit to proceed criteria. Acute medical conditions continue to impact short-notice cancellations. Standardised pathways, improved scheduling, and strengthened perioperative coordination could reduce disruption and improve theatre utilisation.

**Editor’s key points**

• Postponing or cancelling elective surgical procedures can adversely affect the patient experience, clinical outcomes, and operational efficiency. The Postponement and Cancellations in Elective Care (PACE2024) study assessed the incidence and causes of postponement at preoperative assessment and cancellation within 24 h of planned surgery across the NHS in UK.
• Data from 78 NHS trusts from a 7-day survey in 2024 show reduced cancellation rates (now 9.9%) and postponements (8.7%) with improved theatre efficiency (74.7% of lists reported as running efficiently) since the Super-SNAP1 study in 2022. Postponements were most commonly attributable to a need for further investigation, and cancellations were most frequently associated with acute medical conditions and list overruns.
• Because nearly half of postponements involved additional testing or consultations, and acute medical conditions were the main driver of short-notice cancellations, earlier optimisation and robust preoperative assessment to meet fit to proceed criteria are needed. Proactive management of acute medical conditions and patient-initiated reasons for cancellation, optimised theatre scheduling to reduce list overruns, and enhanced preassessment pathways to ensure preparation for surgery could reduce disruption and improve theatre utilisation, with positive impacts on patient experience, workforce, and resource utilisation.

Elective surgical care accounts for around 75% of surgical activity across the UK NHS, playing a key role in enhancing patient quality of life, preventing the progression of life-limiting disease, and supporting broader economic productivity.^1^ Within NHS England, a constitutional standard sets out an expectation that 92% of patients should commence treatment within 18 weeks from the point of referral for elective care pathways.^2^^,^^3^ In February 2022, NHS England initiated a 3-yr plan aimed at recovering elective and cancer care services from the impact of COVID-19.^4^ As of January 2025, ∼6.25 million individuals were awaiting elective care across 7.43 million pathways. Almost half of these, 3.06 million patient pathways, had already exceeded the 18-week target, underscoring the substantial backlog.^5^ A revised elective care reform plan, published in January 2025, sets out the ambition to achieve a return to the constitutional standard by 2028–9. However, the extension of recovery timelines indicates persistent challenges in aligning strategic ambitions with operational capacity, which might require attention to investment, service redesign, or workforce planning to address ongoing pressures.^6^

Among the most impactful disruptions to elective surgical pathways are postponements and cancellations. A study published in 2024 across 16 NHS trusts found a postponement rate of 7.3%, with variation in the incidence by trust ranging from 1.0% to 31.9%.^7^ Of those patients undergoing routine surgery, 85.7% of postponements were attributable to medical reasons and >50% of patients waited >94 days from decision to operate to their preoperative assessment, suggesting there is adequate time in many surgical pathways to allow screening and patient optimisation.^7^

Studies assessing cancellation rates have shown that up to 10% of patients attending for surgery have previously had a cancellation for the same procedure,^8^ with on the day cancellation rates of 5–17.6%.^8^, ^9^, ^10^ In a large UK observational study, nonclinical reasons such as inadequate bed capacity (31%) and insufficient operating theatre capacity (12.7%) accounted for a large proportion of cancellations, with requirement for a postoperative critical care bed independently associated with risk of cancellation (odds ratio [OR], 2.92; 95% confidence interval [95% CI], 2.12–4.02; *P*<0.001).^8^ Patients undergoing cancer surgery (OR, 0.39; 95% CI, 0.22–0.46; *P*<0.001) and expedited surgery (OR, 0.39; 95% CI, 0.27–0.56; *P*<0.001) were less likely to have their procedures cancelled.^8^

Postponement and cancellation events are not merely administrative hurdles; they lead to prolonged patient waits, increased anxiety,^11^ potential clinical deterioration, and substantial inefficiencies in resource allocation, including wasted theatre time and suboptimal staff deployment.^8^ These disruptions can occur at various stages, from preoperative assessment to the immediate pre-surgical period, each presenting distinct causative factors and systemic impacts.

Recognising the significant impact of these disruptions, the Postponement and Cancellations in Elective Care audit 2024 (PACE2024) was conducted. This study aimed to systematically quantify and understand the reasons behind postponements at the preoperative assessment stage and cancellations occurring within 24 h of planned surgery. The postponements aspect of PACE2024 was piloted by McCone and colleagues,^7^ and our methods for the cancellations part of the study were similar to the first Super Sprint National Audit Project (Super-SNAP1).^12^ Super-SNAP1 was a 2-day study that took place in January 2022 across 74% of UK NHS Trusts and Health Boards which collected data on the incidence of cancellations in emergency and elective pathways during the COVID-19 pandemic.^12^ Owing to the similarities in methodology, we compare data from PACE2024 with Super-SNAP1.

## Methods

### Study design and setting

The PACE2024 study was a 7-day service evaluation conducted across NHS trusts in UK. Data were collected prospectively from 08:00, Monday, November 11 to 07:59, Monday, November 18, 2024. The project was a collaborative effort led by the National Institute of Health Research (NIHR) Central London Patient Safety Research Collaboration, NHS England, University College London, and the Centre for Research and Improvement at the Royal College of Anaesthetists (RCoA). As the study was a service evaluation, it did not require formal ethics approval.^13^

### Participants and data collection

NHS trusts were invited to participate *via* the NHS England Perioperative Medicine medical and non-medical lead networks and through the RCoA. Sites were asked to identify a lead for both the postponement and cancellation arms of the audit. Data were collected at a local site level by preoperative assessment non-medical staff, clinicians, and theatre management teams, and submitted through an online platform.^14^ Where a postponement or cancellation took place, more detailed case level data were collected (Supplementary material files 1 and 2). Data on preoperative assessment capacity and planned operating theatre activity were captured using separate structured forms (Supplementary material file 3 and Supplementary file 4). Local investigators were also asked to provide a subjective opinion on whether each operating list ran efficiently or not. Local sites were requested to complete data submissions by December 18, 2024. Data were exported for analysis on January 28, 2025, for postponements and on January 29, 2025, for cancellations.

#### Postponements at preoperative assessment

For those patients with a ‘to come in’ date at the preoperative assessment phase, a postponement was anything that prevented the patient from having surgery on their planned ‘to come in’ date. For those patients without a ‘to come in’ date at the preoperative assessment phase, a postponement was defined by any factor that prevented the patient from having their preoperative assessment finalised as ‘good to go’ within 2 weeks.

#### Cancellations

A cancellation was defined as a decision not to go ahead with surgery taken within the preceding 24 h of the planned date of intervention. When surgery was planned to take place on a Monday, cancellations occurring on the preceding Friday were included.

### Inclusion and exclusion criteria

Only cases that required provision of anaesthetic support in the form of sedation or anaesthesia were included in the audit. Local anaesthetic-only lists and procedures with sedation provided by the performing clinician without anaesthetic support (e.g. endoscopy) were excluded. Emergency and urgent trauma cases (P1 prioritisation) and obstetric indications were also excluded from the service evaluation.

### Data processing and analysis

Data cleaning, processing, and analyses were conducted using R version 4.5.1 (R Foundation for Statistical Computing, Vienna, Austria).^15^ Duplicate submissions were identified and manually removed. Submissions that did not meet the inclusion criteria were also manually removed.

Surgical prioritisation was defined according to the classification system produced by the Federation of Surgical Specialty Associations: P2 classified procedures should be performed within 1 month of decision to operate, P3 procedures within 3 months, and for P4 procedures, a wait of >3 months is deemed acceptable.^16^ Surgical magnitude of cancelled procedures was defined using the Clinical Coding and Scheduling Development (CCSD) classification.^17^

To calculate the national incidence for both cancellations and postponements, only trusts that had submitted both case data and denominator and capacity data were included. CIs around the point estimates were constructed using the bootstrap method, using 1000 bootstrap resamples at NHS trust level.^18^ When showing the national variation in cancellation incidence, sites were excluded if their PACE2024 submissions did not meet ≥80% of NHS England reference data for expected procedures during the data collection period. If no reference data existed, sites in the lowest quartile of denominator number submissions were excluded to reduce bias in incidence related to low case ascertainment.

Local investigators were permitted to select multiple reasons for both cancellations and postponements, meaning the total number of reasons provided could exceed the number of events. Where reasons are presented, the denominator used is the actual reported number of postponements (*n*=2190) or cancellations (*n*=2165) unless otherwise stated. Local data were shared with investigators in the form of standardised reports to facilitate quality improvement at site level.

## Results

Seventy-eight NHS trusts submitted both individual postponement and preoperative assessment capacity data (Supplementary Figure. 1). Overall, 7173 of 22 573 (31.2%) preoperative assessment appointments were conducted virtually, and 15 796 (68.8%) were reported as conducted in person. There was significant variation at trust level, with 74.9% (median; interquartile range [IQR], 48.2–90.2%) of initial preoperative assessment appointments being delivered face-to-face. In a bivariable quasibinomial regression analysis, there was no association between the proportion of initial virtual preoperative assessment appointments and postponement rates at trust level (*β=*−0.0016; se, 0.0039; *P*=0.684).

**Fig 1:**
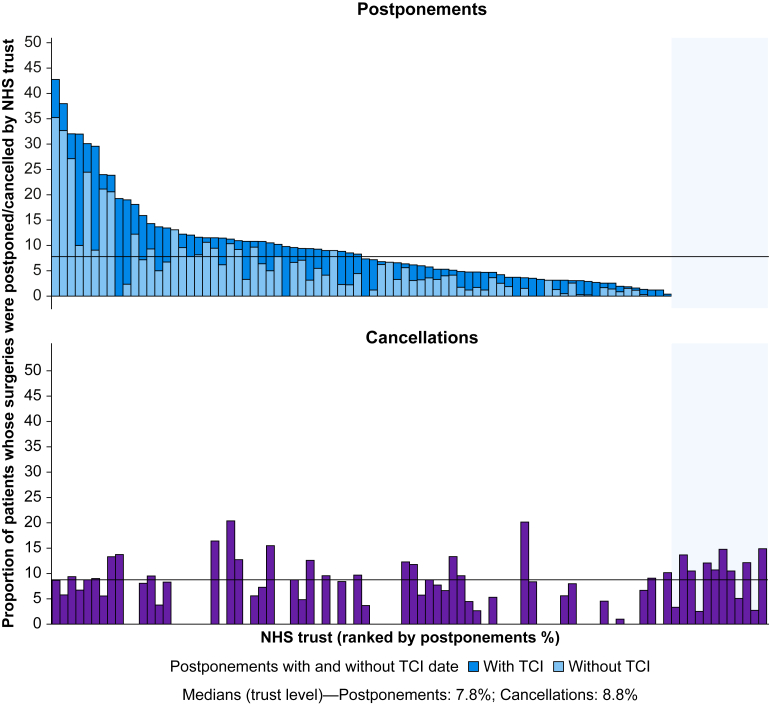
National variation in postponement and cancellation incidence at NHS trust level. Trusts are ranked by the percentage of reported postponed patients compared with reported preassessment capacity. The shaded area to the right of the figure represents trusts that did not submit postponement data. TCI, ‘to come in’.

From 22 573 reported preoperative assessment appointments, 1959 patient surgeries were postponed in the preoperative assessment pathway, resulting in a national postponement incidence of 8.7% (95% CI, 6.9–10.8%). The total number of postponements reported across the 91 participating trusts (including those without preoperative assessment capacity data) was 2190. The reported incidence of postponements at trust level was 7.8% (median; IQR, 3.7–11.6). Fig. 1 shows the variation in postponement incidence by NHS trust, with the proportion of patients whose surgeries were postponed with or without a ‘to come in’ date shown.

Ninety-one NHS trusts provided cancellation data, of which 78 contributed both cancellation and list efficiency data (Supplementary Fig. 1). In these 78 trusts, there were 1974 cancellations out of 19 905 planned procedures, yielding a national cancellation incidence rate of 9.9% (95% CI, 8.7–11.3%) during the study period. The total number of cancellations reported across all participating trusts (including those without list efficiency data) was 2165.

Fig. 1 shows the variation in reported incidence of cancellations amongst NHS trusts, ranging from 1% to 20.4%. The cancellation rate at trust level was 8.8% (median; IQR, 5.6–12.0%). There was a weak, non-significant positive correlation between postponement and cancellation rates across NHS trusts (Spearman’s *⍴*=0.20; 95% CI, −0.07 to 0.46; *P*=0.18). Supplementary Table 1 shows the planned operating theatre activity and preoperative assessment capacity at each NHS trust reporting data to PACE2024.

### Reasons for postponements

For the 2190 reported postponements, 2591 reasons were provided. The top reasons for patient postponements were further preoperative investigation or test required (*n*=595, 27.2%), further assessment or optimisation required (*n*=453, 20.7%), uncontrolled diabetes mellitus (*n*=191, 8.7%), and patients requiring comprehensive geriatric assessment/high-risk anaesthetic clinic or multidisciplinary team (MDT) review (*n*=179, 8.2%; Table 1).

**Table 1:** Reasons for postponement at preoperative assessment. Local investigators were permitted to provide more than one reason for any given postponement. Overall, there were 2591 reasons provided for a total of 2190 postponements reported to the Postponement and Cancellations in Elective Care (PACE2024) study. HbA1c, haemoglobin A1c; TCI, ‘to come in’.

Reason for postponement	*n*	Total postponements (%)
Requires further investigation/preoperative test	595	27.2
Requires any secondary care specialist for assessment/optimisation	453	20.7
Uncontrolled diabetes mellitus (HbA1c >69)	191	8.7
Requires comprehensive geriatric assessment/high-risk anaesthetic clinic or multidisciplinary team (MDT) review	179	8.2
Other	145	6.6
Anaemia requiring correction	126	5.8
Acute infection too close to TCI date	120	5.5
Not enough time to arrange anaesthetic review within the preassessment process (patients that have a TCI date)	108	4.9
Uncontrolled hypertension	96	4.4
No longer requires surgery or patient decided not to proceed	94	4.3
Abnormal blood values for investigation outside of anaemia	77	3.5
Unable to proceed at the specified site because of comorbidity therefore ‘to come in’ postponed (e.g. not suitable for remote elective surgical centre)	72	3.3
Referral back to surgeon for review	71	3.2
Uncontrolled/new atrial fibrillation	60	2.7
Infection control issue	51	2.3
Acute infection/medical condition too close to TCI date	45	2.1
Removed from waiting list as too high risk	42	1.9
Social considerations	34	1.6
No time to stop high-risk medications before TCI date (anticoagulation/antiplatelets/disease modifying drugs)	32	1.5

Of patients whose surgeries were postponed at the preoperative assessment stage, 37.3% had already been given a ‘to come in’ date. Of the 480 P2 patients whose surgeries were postponed (21.9% of total postponements), 204 had a ‘to come in’ date (42.5% of P2 postponements). P3 and P4 prioritisation categories constituted 78.1% (*n*=1710) of postponements, with 613 (35.8%) of these having a ‘to come in’ date.

Of the 2190 postponements, 1144 (52.2%) were planned as day-case procedures, and 1046 (47.8%) were planned on an inpatient basis. Only 343 patients (15.7%) whose surgery was postponed were documented as having gone through an early screening pathway.

Postponement decisions were predominantly made by anaesthetic staff (*n*=1041, 47.5%) although non-medical staff (e.g. preassessment nursing teams) actioned a significant proportion (*n*=890, 40.6%). Surgical teams made the decision in 6.7% of cases, and MDT decisions accounted for 5.1%. When considering postponements in P2 patients who should undergo surgery within 1 month, non-medical staff accounted for 167 postponement decisions (34.8%). Supplementary Figure 2 shows the staff group responsible for postponement decisions by surgical prioritisation category.

For children and young people (<18 yr), 57 postponements were captured (2.6% of total postponements). The main reasons were not enough time for anaesthetic assessment (36.8%) and acute infection close to the ‘to come in’ date (14%).

### Reasons for cancellations

For the 2165 reported cancellations, 2553 reasons were provided. The top three reasons were acute medical conditions (patient or clinician reported, *n*=515, 23.8%), list overruns (*n*=315, 14.5%), and patients not attending for surgery (*n*=243, 11.2%). Table 2 shows the reasons for cancellations within 24 h of planned surgery grouped by categories. Supplementary Table 2 provides more granular detail on reasons given for cancellations.

**Table 2:** Reasons for cancellation within 24 h of planned surgery. Local investigators were permitted to select one or more reasons for a given cancellation. In total, 2553 reasons were provided for 2165 reported cancellations. Reasons for cancellation shown here are grouped by category. Individual reasons given for cancellations at case level are available in Supplementary Table 2.

Reason provided for cancellation	*n*	Total cancellations (%)	Potentially avoidable
Acute medical condition	515	23.8	
List overrun	315	14.5	
Did not attend/was not brought	243	11.2	Yes
Clinical staff unavailable	180	8.3	
Procedure no longer necessary	128	5.9	Yes
Preassessment	127	5.9	
Procedure not wanted	122	5.6	Yes
Emergency admission	111	5.1	
Other	108	5.0	
Pre-existing medical condition	101	4.7	Yes
Equipment unavailable or failed	95	4.4	
Treatment/surgery deferred	93	4.3	Yes
No bed available	77	3.6	
Pre-op guidance not followed	72	3.3	Yes
Undiagnosed condition	55	2.5	
Appointment inconvenient	49	2.3	Yes
Administrative change	47	2.2	
Unsuitable for surgical hub/green site	18	0.8	
Essential support unavailable	7	0.3	
Blood products unavailable	6	0.3	

Potentially avoidable reasons for cancellation, which if recognised 3–5 days before planned surgery could have either been prevented or enabled alternative patients to be booked onto operating lists, were reported for 808 out of the 2165 cancellations (37.3%; see Table 2).

Cancellations related to preassessment processes contributed to 127 (5.9%) cancellations during the data collection period. Of these, lack of investigation and optimisation of existing health conditions accounted for the majority (*n*=108, 5%) (Table 3).

**Table 3:** Cancellations related to preoperative assessment. Individual reasons given for cancellations at case level are available in Supplementary Table 2.

Reason provided for cancellation	Number of times reason given	Total cancellations (%)
Health problem not fully investigated	56	2.6
Appropriate optimisation/follow up not completed	52	2.4
Incomplete paperwork	16	0.7
Appropriate aftercare not arranged	13	0.6
Reasonable adjustments not in place because of disability or mental health issue	3	0.1

Staffing constraints contributed to 191 (8.8%) cancelled procedures. Of the staffing groups, lack of surgeon availability accounted for the greatest proportion of cancellations (*n*=97, 4.5%), followed by anaesthetist availability (*n*=57, 2.6%; Supplementary Table 2).

**Fig 2:**
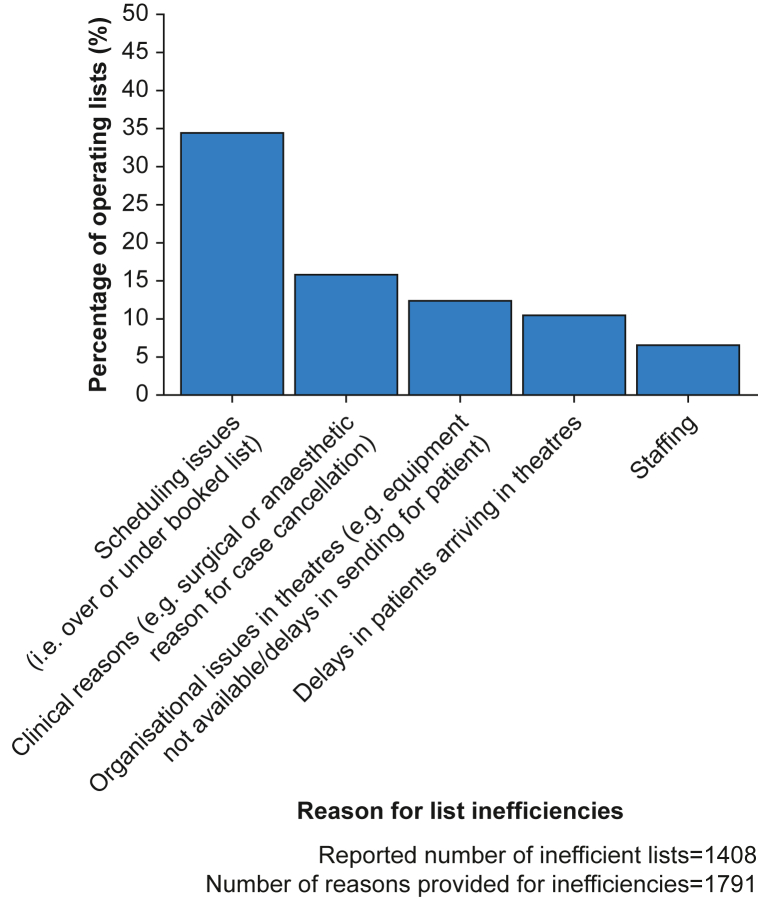
Top five reasons for operating list inefficiencies.

Operating list efficiency data were submitted for 5558 surgical and procedural lists. Of these, 4150 (74.7%) were reported as running efficiently. Fig. 2 shows the top five reasons for inefficient operating lists. The three most reported reasons for inefficiencies were scheduling issues (*n*=616, 34.5%), clinical reasons (*n*=324, 18.2%), and organisational issues in theatres (*n*=223, 12.5%).

## Discussion

The PACE2024 study provides a detailed examination of the rates and reasons for postponements at preoperative assessment and short-notice cancellations of elective surgery in the NHS in UK. With 64.5% of NHS trusts with operating theatres^19^ reporting data, PACE2024 provides a comprehensive national snapshot of the issues facing the elective reform plan, and we highlight three key findings. Firstly, almost 9% of patients had their procedures postponed at the preoperative assessment appointment, and a further 10% were cancelled within 24 h of the planned surgery. This is disruptive for both patients and service, reducing productivity, efficiency, and patient experience. Secondly, almost 40% of last moment cancellations were attributable to potentially avoidable issues, and almost a quarter were attributable to acute medical conditions; at least some of these patients could have been identified in the days approaching their operation date and replaced with patients deemed ready to proceed at short notice. Thirdly, most postponements occurred because patients were not optimally prepared for surgical intervention upon reaching preoperative assessment, requiring further investigations or optimisation.

Patients requiring further specialist assessment (comprehensive geriatric assessment, high-risk anaesthetic clinic, or multidisciplinary review) accounted for 8.2% of postponements (*n*=179). When this is considered alongside postponements owing to lack of time to arrange anaesthesia review (*n*=108, 4.9%), patients no longer wanting or requiring surgery (*n*=94, 4.3%), high-risk patients being removed from the waiting list (*n*=42, 1.9%), and additional short notice cancellations because of patients no longer wanting a procedure (*n*=122, 5.6%), it suggests a potential failure of true shared decision making at the time patients are added to a waiting list. This presents an opportunity to reduce the incidence of both postponements and cancellations by embedding shared decision making earlier in the surgical pathway, ideally right at the time of decision to operate.

Patients attending for preoperative assessment might have been on a surgical waiting list for a year or more or be waiting for time-critical surgery. More than a third of these postponements delayed surgery in patients who had already received a date for their operation, thereby risking poor patient experience, and worse clinical outcomes. Despite continued focus at the national level from initiatives such as the Perioperative Quality Improvement Programme, uncontrolled diabetes mellitus remained the third most common reason for postponement at preoperative assessment.^20^

There was substantial variation in cancellation and postponement rates between trusts (Fig. 1), suggesting opportunities for local improvement and shared learning. Although it is difficult to draw conclusions without understanding specific local pathways, the data underscore the importance of regular review of local performance to identify patterns and inform service improvement plans.

Our findings re-emphasise the importance of reconfiguring patient pathways to ensure that patients are screened for modifiable health conditions as early as possible so that they can be optimised while they wait, and then attend preoperative assessment before a date for surgery is confirmed to undertake a final check of perioperative readiness. In May 2023, NHS England published five core requirements for NHS providers that described how the process of early risk stratification and optimisation should be implemented to improve the care of patients on surgical pathways.^21^ Implementation guidance subsequently promoted the benefit of also screening day-case patients, to identify low-risk patients suitable for a ‘light touch’ pathway, potentially incorporating remote preoperative assessment to free up face-to-face capacity for more complex patients.^22^ Patients identified as low risk might also be suitable to backfill available operating theatre capacity at short notice, such as when planned patients have an acute illness precluding safe surgery and anaesthesia.

Despite these standards and subsequent guidance, the low proportion of patients with postponed procedures in our dataset documented as having undergone early screening (15.7%) suggests that these pathways are yet to be fully embedded across UK. Improved integration within referral pathways, particularly between primary and secondary care as suggested by the recently published NHS 10-year plan, might provide an opportunity for patients to access more diagnostic testing and support with optimisation in community settings before preoperative assessment.^23^

Anaesthetists were the responsible decision maker in the majority of postponements (P2 prioritisation group: *n*=240, 50.0%; P3/4 group: *n*=801, 46.9%). Non-medical staff (e.g. preoperative assessment nursing teams) were the next most common decision makers in both P2 and P3/4 surgical prioritisation groups (P2: *n*=167, 34.8%; P3/4: *n*=723, 42.3%). The postponement decision was taken by the surgical team in only 9.6% of P2 priority patients (*n*=46) and 5.9% of P3/4 priority patients (*n*=101; Supplementary Figure. 2). Given that surgery for P2 classified surgery should be performed within 1 month, it was expected that a greater proportion of this group would have the postponement decision made by an anaesthetist or surgeon compared with P3/4 classified patients, where a wait of more than 1 month is acceptable. More senior oversight of P2 postponements, with clearer multidisciplinary communication and greater surgical involvement, might reduce the number of postponements in this group by supporting clinicians to weigh the risks and benefits of proceeding with or postponing surgery.

The national cancellation incidence of 9.9% is a reduction from the 15.3% reported in Super-SNAP1.^12^ The context for the Super-SNAP1 study was very different to PACE2024, particularly as the NHS was still imposing COVID-19-related restrictions on both patients and hospital staff.^24^ However, the improvement could also reflect benefits from expansion of enhanced care facilities, and the rapid expansion of elective surgical hubs designed to ring-fence beds and minimise the impact of unpredictable emergency pathways on elective capacity.^25^ Despite these relative improvements, our findings indicate that, on average, about one in 10 patients undergoing surgery on any given day does not ultimately undergo their planned procedure. The implications of such cancellations extend beyond the immediate logistical challenges to healthcare services; for the patient, the physical and psychological consequences are considerable. Postponement of surgery can exacerbate underlying symptoms, delay definitive treatment, and heighten anxiety or uncertainty, all of which can adversely affect overall health and wellbeing. These impacts should not be underestimated when evaluating the true burden of surgical cancellations.

The top reasons for cancellations, which include acute medical conditions, list overruns, and patients not attending, underscore areas requiring continued focus. Although the proportion of patients with cancelled procedures owing to acute medical conditions was lower than that reported in Super-SNAP1 (23.8% *vs* 33.4%), it remains the largest single cause.^12^ This highlights the need for robust systems to identify such patients before the day of surgery where possible, enabling timely backfilling of theatre slots. The substantial contribution of list overruns (14.5%) suggests a potential culture of overbooking operating lists to maximise utilisation of available theatre time, which while aiming for efficiency, can lead to patient distress and wasted resources when procedures are cancelled on the day. List overruns also have a significant impact on theatre staff wellbeing. Cancellations owing to list overruns occurred more frequently in longer, more invasive surgery, which disproportionately impacts theatre utilisation.

Despite these potential issues, the reported subjective efficiency of operating lists improved from 64.5% in Super-SNAP1 to 74.7% in PACE2024.^12^ With the increased deployment of electronic health records and theatre scheduling systems, there is the potential to improve efficiency further whilst reducing cancellations because of theatre overruns through the use of granular data to develop predictive models that can more accurately schedule theatre operating lists.^26^

Workforce availability continues to be a significant factor in cancellations, contributing to 8.3% of the total, similar to Super-SNAP1 (9.0%).^12^ Shortages of anaesthetists (2.6%) and surgeons (4.5%) made up the majority of these.

Communication emerges as a critical theme across both postponements and cancellations. Shared decision making, embedded early in the surgical pathway, might help reduce late postponements and short notice cancellations of high-risk patients who often require multiple preassessment visits during their surgical pathway. The number of patients whose procedure was postponed (*n*=94, 4.3%) and cancelled (*n*=250, 11.5%) owing to surgery no longer being required or wanted suggests that earlier shared decision making might not only reduce postponements and cancellations but also improve capacity within the preoperative assessment system if patients decide not to proceed with surgery.

Once a decision is made to proceed, improved preoperative communication with patients whilst awaiting surgery, including a single point of contact and regular check-ins (e.g. 3-monthly touch points for patients on the waiting list), could identify acute illnesses or patient-initiated cancellations (e.g. procedure no longer wanted, inconvenient appointment, non-attendance) earlier. This proactive approach would allow for better backfilling of theatre slots and reduce the administrative burden of rescheduling. Furthermore, enhanced communication between preoperative assessment services and theatre booking teams is essential to reduce cancellations because of incomplete preassessment or scheduling inefficiencies.

### Recommendations to reduce postponements and cancellations

On the basis of our data, we can recommend the following:

1. *Implement early screening for all patients on surgical waiting lists.* Comprehensive early screening of both day-case and inpatient surgical patients should identify both high-risk patients for investigation and optimisation and low-risk patients suitable for streamlined (potential virtual or telephone) preassessment. This will increase capacity for face-to-face assessments where clinically required.
2. *Establish a pool of preassessed, low-risk patients.* Patients deemed low risk should be considered for a flexible ‘fit list’ to backfill late cancellations, particularly those arising from acute medical conditions. Such a system might operate on an opt-in basis, whereby eligible patients agree to being contacted at short notice should capacity become available.
3. *Defer scheduling of surgery until patients are cleared by the preassessment pathway for elective P3 and P4 prioritisation categories.* Date for surgery should only be offered once patients have been deemed fit by preoperative assessment services. Booking and scheduling teams should have visibility of assessment status.
4. *Patients requiring investigation or optimisation should be monitored to ensure that they are not ‘lost’ to the system.* Patients in whom there is a ‘clock stop’ or who remain on a waiting list pending optimisation should be monitored through regular communication. Patients should be contacted at least every 3 months while they wait for surgery, to ensure any changes in health conditions are identified in a timely manner and provide an opportunity for patients to engage in further shared decision making to help avoid cancellation when a procedure is no longer needed.^27^
5. *Adhere to national guidance for preoperative testing.* Investigations should be clinically indicated and aligned with national recommendations to avoid unnecessary delays.^28^, ^29^, ^30^, ^31^
6. *Ensure senior clinician input for P2 surgery postponements.* Elective P2 priority cases should not be postponed or cancelled without the involvement of senior clinicians (e.g. consultant anaesthetist AND surgeon together) to facilitate multidisciplinary shared decision making and prevent avoidable cancellations.
7. *Improve communication via a single point of contact.* A dedicated contact for each patient, such as a perioperative care coordinator, can improve identification of those likely to cancel because of medical issues, unavailability, or a change in preference, enabling timely operating list adjustments. A single point of contact also facilitates timely communication from patients regarding new or ongoing issues whilst awaiting surgery.^22^
8. *Waiting list, booking, and scheduling teams should adopt best practice approaches.* Information should be shared between key teams to identify potential risks for postponements and cancellations in a timely manner. Development of data-driven approaches to theatre scheduling can also reduce the impact of theatre overruns on short notice cancellations.^26^^,^^32^
9. *Assess* *service needs* *and,* *if required,* *expand enhanced care capacity.* Continued development of enhanced care facilities can reduce cancellations linked to critical care capacity. These cancellations typically result in a disproportionate loss of theatre time.
10. *Local and national reporting of high-quality cancellation data.* NHS England has a mandatory data return for theatre cancellations which includes reasons for cancellation. Data submissions are currently varied, and improved local reporting and data quality might facilitate improvement efforts. Postponement and cancellation data should be reviewed at the site and trust level to facilitate pathway optimisation. Data should be fed back to preoperative assessment, anaesthesia, and surgical teams to promote better collaboration including earlier risk assessment and referral for optimisation where appropriate. Surgeons should be supported to improve shared decision making at the time of decision to operate, alongside revised perioperative pathways that enable ongoing engagment, recognising that patients might reconsider their decision to proceed, or their health status might change while awaiting surgery.

### Limitations

As a 7-day snapshot, this study has inherent limitations. Factors influencing cancellations and postponements can vary week by week, and specific local events during the audit period (e.g. new electronic patient record implementation, surgical hub opening) might have exerted unusual operational pressures on individual organisations. Although the participation rate was excellent for this type of study, not all sites and data within those sites will have been captured, which could introduce sampling bias. The methodology also allowed for multiple reasons to be selected for a single event, which can complicate interpretation of primary drivers of postponements and cancellations. Finally, this study did not capture data on patients whose surgeries were not postponed or cancelled. This limits our understanding of the effectiveness of some of the interventions we have discussed, for example, early screening and optimisation, and backfilling of short-notice cancellations of surgery.

More broadly, the study highlights the challenges of capturing perioperative data in a consistent and routine manner. Variability in local information systems, differences in how cancellations are coded, and the administrative burden of manual data collection all pose barriers to reliable surveillance. Although the NHS England theatre productivity data set includes cancellation data, current returns and completion are limited, reducing its utility for benchmarking and longitudinal analysis. These limitations constrain the ability of organisations to monitor the impact of interventions over time and to identify systemic drivers of inefficiency. Emerging initiatives, such as the planned national clinical audit of perioperative care, might provide a framework to embed cancellation and postponement data capture into routine practice at scale. Such an approach could enable more robust, standardised, and longitudinal monitoring, supporting both local quality improvement and national service planning.

### Conclusions

The PACE2024 study offers a comprehensive overview of postponements and cancellations within elective surgical pathways across the NHS in UK, with data reported from 64.5% of NHS trusts with operating theatres. Although a reduction in cancellation rates (now 9.9%; 95% CI, 8.7–11.3%) and an improvement in theatre efficiency (74.7% of lists reported as running efficiently) have been observed since the Super-SNAP1 study, significant challenges persist. Key areas requiring continued focus include the proactive management of acute medical conditions and patient-initiated reasons for cancellation, optimising theatre scheduling to reduce list overruns, and enhancing preassessment pathways to ensure that patients are fully prepared for surgery. The relatively high proportion of postponements occurring in patients with a ‘to come in’ date (37.3%) highlights a critical opportunity for earlier patient optimisation and improved communication between clinical and booking and scheduling teams. Maximising opportunities within the existing workforce to streamline surgical pathways and reduce duplication of administrative processes associated with postponements and cancellations could improve efficiency. Digital transformation enabling routine perioperative data capture from electronic patient records should support high-quality shared decision making, improved understanding of risk, patient involvement in optimisation, and enhanced perioperative productivity across the NHS.

## Authors’ contributions

Study design: JB, EM, AH, SRM, JS, AK

Delivery, support, data analysis, drafting of manuscript: JB, EM, AH, SRM, JS

Site recruitment: JY, AF, JH, LSC, AK

Study delivery, administrative support: JY, AF, JH, LSC

Study support: EW, IM, MB, LC, SH, JK, EK, GS

Revision of manuscript: all authors

## Funding

This work was supported by the National Institute for Health and Care Research (NIHR) Central London Patient Safety Research Collaborative. No specific funding was received for this study; however, the collaborative's infrastructure supported the conduct of the work. Some authors were supported through salaried time funded by the NIHR through the Patient Safety Research Collaborative.

## Declarations of interest

JS, EM, and SRM all provide clinical leadership to perioperative care at NHS England on a secondment basis. The other authors declare that they have no conflicts of interest.

## Figures and Tables

**Figure:**
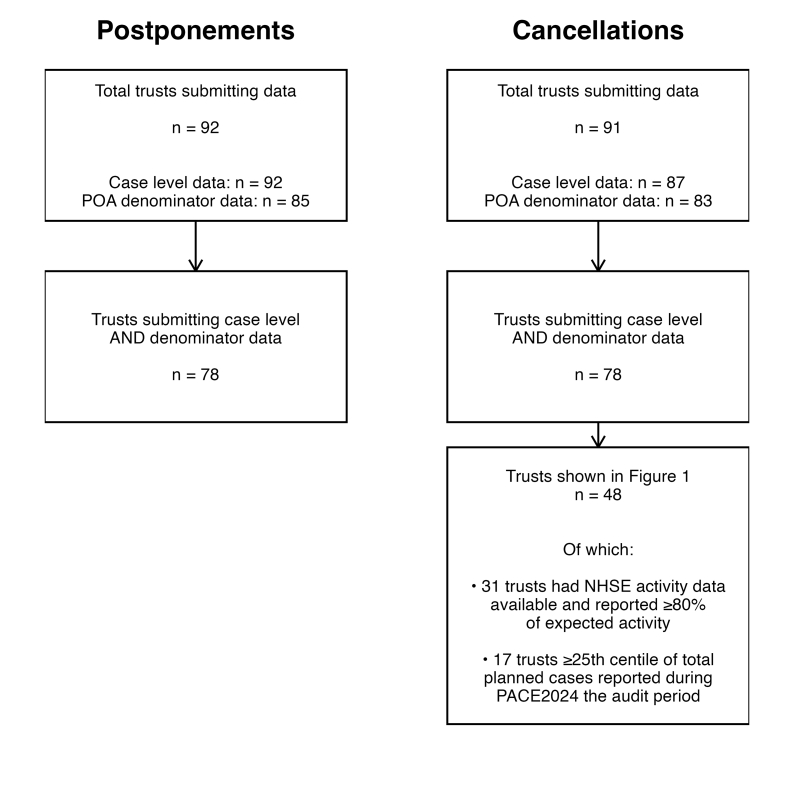


**Figure:**