# Supplementary material for: The Postponement and Cancellations in Elective Care study: a national evaluation of case postponements and cancellations in elective surgical pathways
Source: Br J Anaesth. 2026 Apr 24;136(6):1925–34. doi: 10.1016/j.bja.2026.01.046 (PMC13197895; doi:10.1016/j.bja.2026.01.046)
Supplement: Multimedia component 1 [file mmc1.pdf]

PACE 2024 Form A: Elective postponements (one form per postponement)

**Trust name:**					
**Hospital/site name:**					
**TCI date:** dd/mm/yy					
**Has patient been through an early risk, screening and optimisation pathway prior to POA? (tick)**					
Yes		No		Don't know	
**Age (tick)**					
<18 years		≥18 years			
**Planned as day-case or inpatient? (tick)**					
Day-case		Inpatient			
**Surgical magnitude? (tick)**					
Minor		Intermediate		Major, major+/complex	
**Surgical urgency? (tick/circle)**					
P2 (<1 month)		P3 (<3 months)		P4 (>3 months)	
**Surgical specialty (tick)**					
Breast		Gynaecology		Plastics/reconstruction	
Cardiac		Head & Neck		Thoracics	
Colorectal		Hepatobiliary		Upper GI	
Dentistry		Interventional Radiology/Imaging		Urology	
Endocrine		Maxillo-facial		Vascular	
ENT		Neurosurgery		Other, please specify:	
Gastroenterology		Orthopaedics			
General		Paediatrics			

**Reason(s) for postponement (please tick all that apply)**			
Uncontrolled diabetes (HBA1C >69)		Infection control issue	
Uncontrolled hypertension		No time to stop high risk medications before TCI date (anticoagulation/antiplatelet/DMARDs)	
Uncontrolled/new Atrial Fibrillation		Unable to proceed at the specified site due to comorbidity therefore TCI postponed e.g. not suitable for remote elective surgical centre)	
Anaemia that requires correcting		Social considerations	
Abnormal blood values for investigation outside of anaemia		No longer requires surgery or patient decided not to proceed.	

Requires any secondary care specialist for assessment/optimisation		Referral back to surgeon for review	
Requires further investigation/ pre-operative test		Not enough time to arrange anaesthetic review within the pre-assessment process (with patients that have a TCI date)	
Requires comprehensive geriatric assessment, high risk anaesthetic clinic or multidisciplinary team (MDT) review		Acute infection too close to TCI date	
Removed from waiting list – too high risk			
Other, please specify:			
**Decision to postpone made by (tick)**			
Anaesthetist		Non-medical staff	
Surgeon		MDT decision	
