# Supplementary material for: The Postponement and Cancellations in Elective Care study: a national evaluation of case postponements and cancellations in elective surgical pathways
Source: Br J Anaesth. 2026 Apr 24;136(6):1925–34. doi: 10.1016/j.bja.2026.01.046 (PMC13197895; doi:10.1016/j.bja.2026.01.046)
Supplement: Multimedia component 2 [file mmc2.pdf]

PACE 2024 Form 1: Cancellations (one form per cancelled patient)

**Trust name:**										
**Hospital/site Name:**										
**Date of elective list from which patient was cancelled:** ___/11/2024										
**Age (tick)**										
<18 years				>18 years						
**Planned as day-case or inpatient? (tick)**										
Day-case			Inpatient							
**Did the patient go through a preoperative assessment process before the day of surgery? (tick)**										
Yes			No			Don't know				
**Surgical magnitude? (tick)**										
Minor			Intermediate			Major, major+/complex				
**Surgical urgency? (tick/circle)**										
P2 (<1 month)			P3 (<3 months)			P4 (>3 months)				
**Indication for treatment (tick/circle)**										
Cancer		Cardiac		Vascular		Other				
**Surgical specialty (tick)**										
Breast		Gynaecology		Plastics/reconstruction						
Cardiac		Head & Neck		Thoracics						
Colorectal		Hepatobiliary		Upper GI						
Dentistry		Interventional Radiology/Imaging		Urology						
Endocrine		Maxillo-facial		Vascular						
ENT		Neurosurgery		Other, please specify:						
Gastroenterology		Orthopaedics								
General		Paediatrics								

**Reason for cancellation (please tick all that apply)**			
Pre-existing medical condition		Clinical Staff Unavailable - Surgeon	
Undiagnosed condition		Clinical Staff Unavailable - Anaesthetist	
Acute medical condition – related to COVID		Clinical Staff Unavailable – scrub practitioner	
Acute medical condition – all other		Clinical Staff Unavailable – Anaesthetic practitioner	
Procedure no longer necessary		Clinical Staff Unavailable – Recovery Practitioner	
Unsuitable for surgical hub/green site		Equipment unavailable or failed	
Inadequate Pre-assessment - Incomplete paperwork		Administrative Change - Booked to incorrect session	
Inadequate Pre-assessment - health problem not fully investigated		Administrative Change - Patient brought forward	
Inadequate Pre-assessment - Appropriate optimisation/follow up not completed		Essential support unavailable – Perfusionist/Cell Salvage	
Inadequate Pre-assessment - Appropriate aftercare not arranged		Essential support unavailable - Radiology	
Inadequate Pre-assessment - Reasonable adjustments not in place due to disability or mental health issue		Essential support unavailable – Manufacturer rep	
Treatment/Surgery deferred		Essential support unavailable - Interpreter	
No Bed Available - General / Ward		Preoperative guidance not followed	
No Bed Available - ITU/HDU		Appointment inconvenient	
No Bed Available - ITU/HDU		Unfit for procedure	
No Bed Available - Paediatric		Procedure not wanted	
No Bed Available - Maternity		Did not attend/was not brought in	
Emergency Admission		Industrial action (any staff group)	
List Overrun - Booking error		Blood products unavailable	
List Overrun - Complexity of procedures		Other, please specify:	
List Overrun - Theatre inefficiencies			
List Overrun - Other reason			
