# Supplementary material for: The Postponement and Cancellations in Elective Care study: a national evaluation of case postponements and cancellations in elective surgical pathways
Source: Br J Anaesth. 2026 Apr 24;136(6):1925–34. doi: 10.1016/j.bja.2026.01.046 (PMC13197895; doi:10.1016/j.bja.2026.01.046)
Supplement: Multimedia component 3 [file mmc3.pdf]

PACE 2024 Form B - Weekly POA overview (one form per site per week)

**Trust/hospital name:**	
**Site name:**	
**Date of completion:**	
	Total number this week
**All initial reviews of patients in preassessment during audit week (i.e. total capacity)***	
Completed initial virtual/telephone reviews	
Completed initial in person appointments	
Initial appointments cancelled/deferred by patient and not otherwise used (including DNAs)	
Initial appointments cancelled by preassessment service and not otherwise used	
Available appointments for new POA patients not accounted for above (i.e. unused appointments)	
Number of dedicated anaesthetic POA sessions	

*(do not include appointments solely for investigations, e.g. phlebotomy)
