# Supplementary material for: The Postponement and Cancellations in Elective Care study: a national evaluation of case postponements and cancellations in elective surgical pathways
Source: Br J Anaesth. 2026 Apr 24;136(6):1925–34. doi: 10.1016/j.bja.2026.01.046 (PMC13197895; doi:10.1016/j.bja.2026.01.046)
Supplement: Multimedia component 4 [file mmc4.pdf]

PACE 2024 Form 2: Elective list cancellations and efficiency (one form per operating/interventional list)

**Trust name:**	
**Hospital/site name:**	
**Date of list:**	

**Total number of patients planned to undergo anaesthetic intervention on the list today (at 8am on day prior to scheduled list)**	
……………… patients	
**Actual number of patients who underwent anaesthetic intervention on the list today**	
……………… patients	Please fill out the table below to indicate **the number of patients** treated in each category of urgency and day case vs, inpatient care.

	P1	P2 Cancer	P2 Non-cancer	P3	P4
Adult day case					
Adult inpatient					
Paediatric day case					
Paediatric inpatient					

In the opinion of the surgical and anaesthetic team, was the full time allocated for surgery and anaesthesia on this list used as efficiently as possible? (tick)

Yes			No	

If the list was not used as efficiently as possible, please select all reasons why this was

Uncertainty over hospital bed availability		Delays in patients arriving in theatres	
Uncertainty over critical care or enhanced perioperative care bed availability		Delays in patients being able to leave theatres (e.g. recovery full/lack of physical ICU bed availability/mandated removal of all airway devices in theatre)	
Infection control issues (e.g. cleaning theatres, mandated delays between patients, unavailability of infection control test results)		Organisational issue in theatres (e.g. equipment not available, delays in sending for patient)	
Clinical reasons (e.g. surgical or anaesthetic reason for case cancellation)		Scheduling issues (i.e. over or under booked list)	
Prioritisation of emergency cases over scheduled elective patients		Staffing *(see next section)*	
Other, please specify:			
