# Supplementary material for: The Postponement and Cancellations in Elective Care study: a national evaluation of case postponements and cancellations in elective surgical pathways
Source: Br J Anaesth. 2026 Apr 24;136(6):1925–34. doi: 10.1016/j.bja.2026.01.046 (PMC13197895; doi:10.1016/j.bja.2026.01.046)
Supplement: Multimedia component 5 [file mmc5.docx]

#### Supplementary tables

Supplementary table 1: Reported operating theatre activity and preoperative assessment appointment capacity at NHS trust level

| NHS trust | Reported planned operating theatre activity  (n operations =) | Reported POA capacity  (n appointments =) |
| --- | --- | --- |
| 1 | 606 | 105 |
| 2 | 343 | 379 |
| 3 | 153 | 240 |
| 4 | 293 | 100 |
| 5 | 526 | 315 |
| 6 | 324 | 44 |
| 7 | 161 | 175 |
| 8 | 374 | 378 |
| 9 | 80 | 26 |
| 10 | 115 | 84 |
| 11 |  | 204 |
| 12 | 531 | 611 |
| 13 | 284 | 300 |
| 14 | 247 | 263 |
| 15 | 143 | 104 |
| 16 | 154 | 160 |
| 17 |  | 418 |
| 18 |  | 449 |
| 19 | 158 | 377 |
| 20 | 149 | 190 |
| 21 | 219 | 330 |
| 22 |  | 437 |
| 23 | 186 | 222 |
| 24 | 158 | 119 |
| 25 | 110 | 212 |
| 26 | 542 | 425 |
| 27 | 150 | 204 |
| 28 | 161 | 180 |
| 29 | 113 | 509 |
| 30 |  | 102 |
| 31 | 152 | 167 |
| 32 | 407 | 340 |
| 33 | 465 | 64 |
| 34 | 89 | 366 |
| 35 | 199 | 289 |
| 36 |  | 156 |
| 37 | 199 | 261 |
| 38 | 173 | 269 |
| 39 | 659 | 361 |
| 40 | 164 | 191 |
| 41 |  | 167 |
| 42 | 47 | 192 |
| 43 | 50 | 400 |
| 44 |  | 243 |
| 45 | 357 | 374 |
| 46 | 367 | 357 |
| 47 | 122 | 215 |
| 48 | 200 | 225 |
| 49 | 246 | 243 |
| 50 | 437 | 808 |
| 51 | 202 | 291 |
| 52 | 415 | 226 |
| 53 | 133 | 84 |
| 54 | 296 | 294 |
| 55 |  | 171 |
| 56 | 301 | 194 |
| 57 |  | 520 |
| 58 | 241 | 374 |
| 59 | 198 | 243 |
| 60 | 379 | 988 |
| 61 | 233 | 198 |
| 62 | 56 | 180 |
| 63 |  | 219 |
| 64 |  | 223 |
| 65 | 162 | 228 |
| 66 | 210 | 197 |
| 67 | 358 | 428 |
| 68 | 303 | 417 |
| 69 |  | 224 |
| 70 | 346 | 235 |
| 71 |  | 434 |
| 72 | 294 | 468 |
| 73 |  | 327 |
| 74 | 105 | 441 |
| 75 | 193 | 300 |
| 76 | 165 | 85 |
| 77 |  | 978 |
| 78 | 309 | 256 |
| 79 | 115 | 90 |
| 80 | 235 | 396 |
| 81 | 206 |  |
| 82 | 396 |  |
| 83 | 464 |  |
| 84 | 635 |  |
| 85 | 375 |  |
| 86 | 304 |  |
| 87 | 192 |  |
| 88 | 315 |  |
| 89 | 265 |  |
| 90 | 278 |  |

Supplementary table 2: Reasons for cancellation within 24-hours of surgery

| Classification of cancellation | Reason for cancellation | Number of times reason reported | Proportion of total cancellations (%) |
| --- | --- | --- | --- |
| Clinical | Acute medical condition - all other | 287 | 13.3 |
| Patient | Did not attend/was not brought | 243 | 11.2 |
| Patient | Unfit for procedure | 238 | 11.0 |
| Non-Clinical | List Overrun - Complexity of procedures | 204 | 9.4 |
| Clinical | Procedure no longer necessary | 128 | 5.9 |
| Patient | Procedure not wanted | 122 | 5.6 |
| Non-Clinical | Emergency Admission | 111 | 5.1 |
| Other | Other | 108 | 5.0 |
| Clinical | Pre-existing medical condition | 101 | 4.7 |
| Non-Clinical | Clinical Staff Unavailable - Surgeon | 97 | 4.5 |
| Non-Clinical | Equipment unavailable or failed | 95 | 4.4 |
| Clinical | Treatment/Surgery deferred | 93 | 4.3 |
| Non-Clinical | List Overrun - Other reason | 82 | 3.8 |
| Patient | Pre-op guidance not followed | 72 | 3.3 |
| Non-Clinical | Clinical Staff Unavailable - Anaesthetist | 57 | 2.6 |
| Clinical | Inadequate Pre-assessment - health problem not fully investigated | 56 | 2.6 |
| Clinical | Undiagnosed Condition | 55 | 2.5 |
| Clinical | Inadequate Pre-assessment - Appropriate optimisation/follow up not completed | 52 | 2.4 |
| Patient | Appointment inconvenient | 49 | 2.3 |
| Non-Clinical | No Bed Available - General / Ward | 43 | 2.0 |
| Non-Clinical | Administrative Change - Booked to incorrect session | 40 | 1.8 |
| Non-Clinical | List Overrun - Booking error | 35 | 1.6 |
| Non-Clinical | List Overrun - Theatre inefficiencies | 24 | 1.1 |
| Non-Clinical | No Bed Available - ITU/HDU | 24 | 1.1 |
| Clinical | Unsuitable for Surgical Hub/Green site | 18 | 0.8 |
| Clinical | Acute medical condition - related to COVID | 16 | 0.7 |
| Clinical | Inadequate Pre-assessment - Incomplete paperwork | 16 | 0.7 |
| Non-Clinical | Clinical Staff Unavailable - Scrub practitioner | 15 | 0.7 |
| Clinical | Inadequate Pre-assessment - Appropriate aftercare not arranged | 13 | 0.6 |
| Non-Clinical | Clinical Staff Unavailable - Anaesthetic practitioner | 11 | 0.5 |
| Non-Clinical | Clinical Staff Unavailable - Recovery practitioner | 11 | 0.5 |
| Non-Clinical | No Bed Available - Paediatric | 8 | 0.4 |
| Non-Clinical | Administrative Change - Patient brought forward | 7 | 0.3 |
| Non-Clinical | Blood products unavailable | 6 | 0.3 |
| Non-Clinical | No Bed Available - PACU/POCU/Enhanced Care | 6 | 0.3 |
| Non-Clinical | Essential support unavailable - Radiology | 5 | 0.2 |
| Clinical | Inadequate Pre-assessment - Reasonable adjustments not in place due to disability or mental health issue | 3 | 0.1 |
| Non-Clinical | Essential support unavailable - Interpreter | 2 | 0.1 |

*Notes: Local investigators were permitted to select more than one reason for each cancellation. Therefore, when rows in Supplementary Table 2 are combined into groups to produce Table 2 (main body) the group n() and % may not equal the sum of all individual elements. For example, if for a given cancellation the surgeon and anaesthetist was unavailable – in the above table these are presented individually, but in Table 2 this would only count as one “staff availability” cancellation.*

Supplementary table 3: Cancellations due to list overruns and surgical magnitude

| Surgical magnitude | Number of cancellations related to list overruns | Percentage of cancellations due to list overruns | Total number of patients cancelled | Percentage of total cancellations |
| --- | --- | --- | --- | --- |
| Intermediate | 147 | 46.7 | 1001 | 46.2 |
| Major, Major+ or Complex | 89 | 28.3 | 382 | 17.6 |
| Minor | 79 | 25.1 | 782 | 36.1 |
